# Regulatory motifs found in the small heat shock protein (sHSP) gene family in tomato

**DOI:** 10.1186/s12864-018-5190-z

**Published:** 2018-12-11

**Authors:** Debora Arce, Flavio Spetale, Flavia Krsticevic, Paolo Cacchiarelli, Javier De Las Rivas, Sergio Ponce, Guillermo Pratta, Elizabeth Tapia

**Affiliations:** 1 IICAR-CONICET, Facultad de Ciencias Agrarias, Universidad Nacional de Rosario, Campo Experimental Villarino, Zavalla, S2125ZAA Argentina; 2 CIFASIS - CONICET, Ocampo y Esmeralda, Rosario, S2000EZP Argentina; 3 Cancer Research Center CiC-IBMCC, CSIC/USAL, Campus Miguel de Unamuno s/n, Salamanca, 37007 Spain; 4 GADIB-FRSN-UTN, Colon 332, San Nicolas, B2900LWH Argentina; 5 Faculty of Exact Sciences, Engineering and Surveying, Av. Pellegrini 250, Rosario, S2000BTP Argentina

**Keywords:** sHSP, Heat shock protein, Regulatory motif, TFBS, HSE, Tomato

## Abstract

**Background:**

In living organisms, small heat shock proteins (sHSPs) are triggered in response to stress situations. This family of proteins is large in plants and, in the case of tomato (*Solanum lycopersicum*), 33 genes have been identified, most of them related to heat stress response and to the ripening process. Transcriptomic and proteomic studies have revealed complex patterns of expression for these genes. In this work, we investigate the coregulation of these genes by performing a computational analysis of their promoter architecture to find regulatory motifs known as heat shock elements (HSEs). We leverage the presence of sHSP members that originated from tandem duplication events and analyze the promoter architecture diversity of the whole sHSP family, focusing on the identification of HSEs.

**Results:**

We performed a search for conserved genomic sequences in the promoter regions of the sHSPs of tomato, plus several other proteins (mainly HSPs) that are functionally related to heat stress situations or to ripening. Several computational analyses were performed to build multiple sequence motifs and identify transcription factor binding sites (TFBS) homologous to HSF1AE and HSF21 in *Arabidopsis*. We also investigated the expression and interaction of these proteins under two heat stress situations in whole tomato plants and in protoplast cells, both in the presence and in the absence of heat shock transcription factor A2 (HsfA2). The results of these analyses indicate that different sHSPs are up-regulated depending on the activation or repression of HsfA2, a key regulator of HSPs. Further, the analysis of protein-protein interaction between the sHSP protein family and other heat shock response proteins (Hsp70, Hsp90 and MBF1c) suggests that several sHSPs are mediating alternative stress response through a regulatory subnetwork that is not dependent on HsfA2.

**Conclusions:**

Overall, this study identifies two regulatory motifs (HSF1AE and HSF21) associated with the sHSP family in tomato which are considered genomic HSEs. The study also suggests that, despite the apparent redundancy of these proteins, which has been linked to gene duplication, tomato sHSPs showed different up-regulation and different interaction patterns when analyzed under different stress situations.

**Electronic supplementary material:**

The online version of this article (10.1186/s12864-018-5190-z) contains supplementary material, which is available to authorized users.

## Background

Heat shock proteins (HSPs) are a family of stress-inducible molecular chaperones, ubiquitously present in all forms of life, which main function is to prevent unspecific protein aggregation during stress response [1]. HSPs group into five classes according to weight, HSP100, HSP90, HSP70, HSP60, and small heat-shock proteins (sHSPs), of characteristic low molecular weight (12-40 kDa) [2]. In plants, sHSPs play an important role by contributing to maintain cellular homeostasis during physiological stress [3–5]. Although heat stress is well-known stimuli triggering the induction of sHSPs, it is not the only one. In particular, in both *Arabidopsis* and tomato plants, sHSPs are also induced during development [6, 7] and fruit maturation [8–11], suggesting the existence of a chaperone-dependent regulatory network associated with these processes to maintain cellular homeostasis. Remarkably, many plant genes involved in environmental stress responses seem to be the product of duplication events [12, 13]. This is particularly true for the tomato sHSP gene family, for which roughly half of the members, 17 out of a total of 33, seem to have arise from tandem duplication events [14, 15]. We note, however, that despite their high degree of sequence identity, tandem duplicated sHSP genes, can exhibit diverse patterns of gene expression [14, 16–18], a feature that complicates their functional characterization.

In this paper, we present a study of the sHSP gene family in tomato from the perspective of their sequence conserved promoter architecture related to heat shock response and fruit ripening; and their expression up-regulation and participation in protein-protein interaction networks in the presence or the absence of the heat shock transcription factor A2 (HsfA2), that is a main regulator of HSPs in tomato [19]. Towards this end, we wonder to what extent deviations from the expected nearby promoter architecture of sHSP genes in tomato can explain differences in their patterns of expression during stress response. In this way, we naturally expect the presence of heat shock elements (HSEs) [20, 21], motifs of a rather complex structure, known to be present in the promoter region of HSP genes and some other heat responsive genes [22], which provide DNA binding sites for heat shock factors (Hsfs) [23]. HSEs are gapped palindromic motifs of a modular structure composed by head (GAA) and tail (TTC) subsequences allowing gaps (nn) between them (GAAnnTTC) [24]. Aiming at a precise characterization of HSEs in the promoter region of sHSP genes from tomato, a Position Weight Matrix (PWM) approach supported by a careful data curation process and stringent quality controls [25] was devised. Having characterized the HSE presence in the sHSP gene family, we analyze the up-regulation of different sHSP genes during heat shock. For this purpose, we evaluate the interactions among proteins of co-expressed genes, focusing on sHSP genes, using public available heat stress experiment datasets of tomato.

## Methods

To gain insight into the difficulties of modeling the presence of the gapped HSE motif in the promoter regions of the 33 sHSP genes in tomato, we first considered the DRIMust motif discovery tool [26] allowing the identification of over-represented motifs, including the possibility of gapped ones. A HSE PWM (HSF3) was obtained with DRIMust, but it failed to detect the HSE motif in the promoter regions of two sHSPs that were used as controls (Solyc03g113930 and Solyc08g062450) [27], probably due to the difficulties of modeling palindromic gapped motifs. To overcome DRIMust limitations, we augmented HSE data using the promoter region of heat responsive and ripening genes, and considered the alternative use of the XXmotif [28] matrix-building tool guided by its unique ability to deal with both gap and palindromic constraints and its focus on the optimization of the statistical significance of candidate PWMs.

### HSE dataset

DNA regions of 1000 bp immediately upstream of the TSS were extracted from two independent groups, named clusters 10s and 5t of differentially expressed and co-regulated genes during heat stress and fruit ripening that were originally reported in [27]. Members of cluster 5t, specific to fruit ripening, include target tandem duplicated sHSP genes of chromosome 06 (Solyc06g076520, Solyc06g076560, Solyc06g076570) described previously [14, 29], and HsfA2c (Solyc06g053950). Members of cluster 10s, specific to heat stress, include HsfA2 (Solyc08g062960) and HSP70 (Solyc06g076020). Both clusters share the coactivator MBF1c (Solyc01g104740), known to control heat-response regulon in *Arabidopsis*[30] (Additional file 1).

### Construction of HSE PWMs

Owing to its k-mer seed-based approach allowing the presence of gaps within motifs, the XXmotif tool was used to obtain HSE PWMs in tomato (Additional file 1). The XXmotif tool was parametrized with a pre-calculated background model with Markov order = 2 built from *S. lycopersicum* cv Heinz 1706 (SL2.50.29) genome obtained with the conversion utility tool in the RSAT platform [31]; model: zoops (zero, one or multiple expected occurrences of motifs per sequences); similarity threshold for merging motifs/PWMs:Medium; pseudocounts:10%; max number of gaps in 5-mer seed:2; start search with seed patterns:(3+3)-mer palindromes; use only first N sequences of alignment:ALL; XXmasker:YES. HSE PWMs obtained from clusters 5t and 10s were analyzed with the TOMTOM motif comparison tool [32] and a HSF1AE and HSF21 (*q*-value ≤ 0.005) classification from *Arabidopsis* DAP-Seq [33] experiments was obtained (Additional file 1).

### Quality control of PWMs

**Fig. 1:**
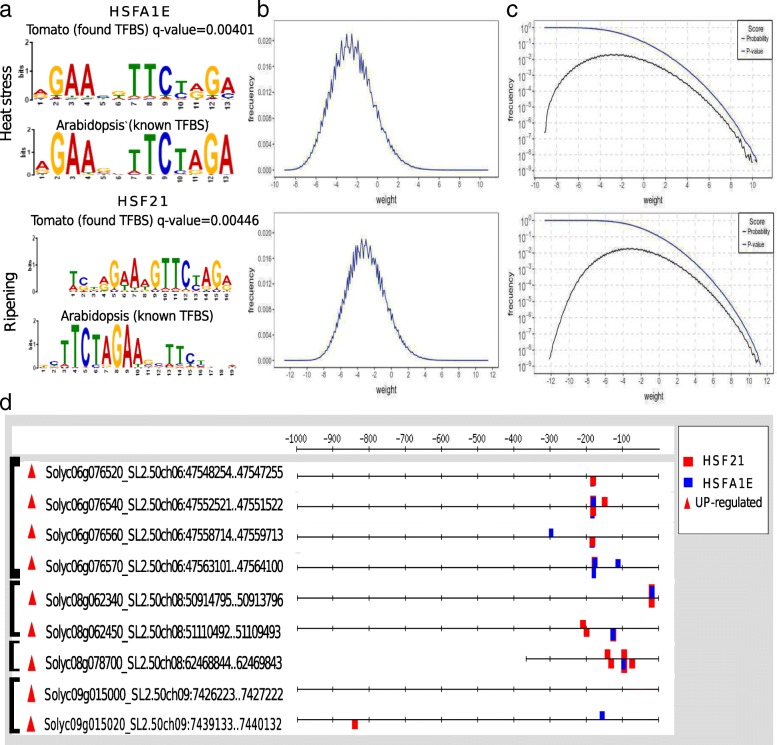
Regulatory motifs found in tomato (*S. lycopersicum cv Heinz 1706*) corresponding to HSFA1E and HSF21 TFBS associated to heat stress and ripening. (**a**) Logo of the position weight matrix (PWM) of the found TFBS in tomato and the corresponding known in *Arabidopsis* for heat stress (upper panels) and for ripening (lower panels). (**b**) Theoretical distribution of weight scores obtained during the construction of the PWMs of tomato and (**c**) corresponding decreasing cumulative distribution functions (dCDF) for the found TFBS in tomato: HSFA1E (upper panels) and HSF21 (lower panels), respectively. (**d**) Location of the HSFA1E (blue-squares) and HSF21 (red-squares) motifs found in the promoter regions (i.e. upstream) of 9 sHSPs of tomato. These genes are also up-regutaled (UP, red triangles) during ripening [14] and are arranged in tandem in chromosomes 06, 08 and 09

To estimate the ability of PWMs to distinguish true binding sites from genome background, their quality was assessed with the methodology described in [25]. For each PWM, the theoretical distribution of weight scores was estimated with the RSAT matrix-distrib tool (Fig. 1b). Corresponding decreasing cumulative distribution functions (dCDF), indicating the probability of observing by chance weight scores equal or higher than a given value, were used to estimate a suitable *p*-value cutoff. Taking into account the dCDFs for HSFA1E and HSF21, a *p*-value cutoff of 0.0001, for weight scores greater than 6, was selected (Fig. 1c and Additional file 1).

### HSE PWM scanning of sHSP gene family promoters

HSFA1E and HSF21 PWMs obtained with XXmotif tool (Fig. 1a) were used to scan the promoter regions of the sHSP gene family [14] (Additional file 1) using the RSAT matrix-scan analysis tool [34]. Matrix-scan analysis was performed with an organism specific background model, Markov order = 2, a cutoff *p*-value = 0.0001 (as previously described), and all the other parameters with default values.

### PPIs analysis

The Uniprot [35] protein IDs from transcriptionally [27] and traductionally [36] active co-expressed genes described in heat stressed (i) tomato wild type plants, and (ii) tomato RNAi protoplasts depleted in HsfA2 [27] (Additional file 2), were used as inputs for STRING [37], a quality controlled functional association network database, according to documentation and user manuals [38]. Five channels of evidence, “experiments” (significant protein interaction datasets, gathered from other protein-protein interaction databases), “database” (significant protein interaction groups, gathered from curated databases), “textmining” (significant protein interaction groups, extracted from the abstracts of scientific literature), “co-expression” (genes that are co-expressed in the same or in other species) and “fusion” (individual gene fusion events per species), were used for computing association scores with default (medium level) score thresholds. Channels “neighborhood” and “occurrence” were not considered observing that they were mainly associated to prokaryotic genomes, or for orthology assignment, which would require further analysis of the sHSP gene family.

## Results

### HSEs in the regulatory region of sHSP genes

Uncovering HSEs in promoters of *S. lycopersicum* cv Heinz 1706 sHSP gene family can shed light on their functional characterization. As expected, HSEs indeed appear in the regulatory region of most sHSP up-regulated genes during tomato fruit ripening (Additional file 1). Conversely, regulatory regions of down-regulated, not-differentially expressed or not-expressed sHSP genes are almost depleted of HSEs during fruit ripening. Specifically, HSEs are observed in cytosolic sHSPs located in chromosome 6 (Solyc06g076520, Solyc06g076540, Solyc06g076560 and Solyc06g076570) and chloroplastic sHSPs located in chromosome 8 (Solyc08g062340 and Solyc08g062450), which members are all up-regulated during fruit ripening (Fig. 1d). In addition, HSEs heavily populates the unique mitochondrial up-regulated sHSP gene (Solyc08g078700) co-localized with other two sHSP genes located in chromosome 8 (Solyc08g078710 and Solyc08g078720). An exception to the apparent rule of HSE presence and sHSP up-regulation is observed in chromosome 09. In this case, two tandemly arrayed sHSP genes are up-regulated but just one of them (Solyc09g015020) shows the HSE presence while the other (Solyc09g015000) seems to be completely depleted from HSEs, at least in their most frequent variants (Fig. 1d).

### Heat shock protein networks related to HsfA2

**Fig. 2:**
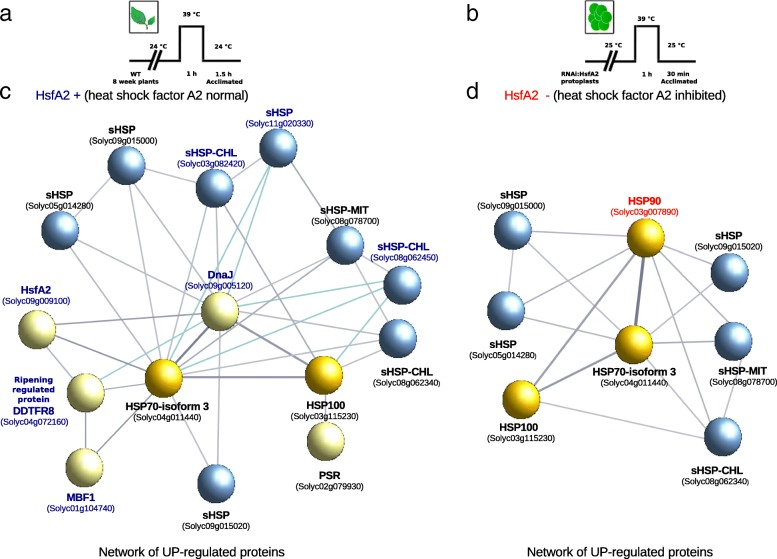
Protein networks built with the sHSPs and HSPs that are up-regulated (i.e. overexpressed) as a response to heat stress treatment. (**a**) Heat stress induced in wild type (WT) tomato plants (where HsfA2 is present); (**b**) heat stress induced in tomato protoplasts (where HsfA2 was inhibited using RNAi). The two networks correspond to the two conditions tested: i.e., (**c**) tomato plants in the presence of HsfA2 (blue label) and (**d**) tomato protoplasts in the absence of HsfA2 (red label). In the networks: nodes are proteins and edges interactions. The proteins included in the networks were HSPs, sHSPs and related proteins up-regulated after heat stress: HSP70-90-100s (yellow nodes), sHSPs (blue nodes) and other heat stress related proteins (light yellow nodes). Colored labels indicate specific proteins dependent on the presence (blue) or absence (red) of HsfA2. The interactions were derived from the known functional or physical associations between proteins

Exception to the HSE presence in promoters of up-regulated sHSP genes during stress, including fruit ripening, rises the question about its actual importance for triggering expression of sHSP genes. We wonder if Solyc09g015000 may be behave like MBF1c, known to be a key factor during thermoregulation in *Arabidopsis*, which shows the absence of HSE in its promoter region. In order to elucidate Solyc09g015000 function, we considered possible protein interactions through a protein network analysis of up-regulated genes during heat stress conditions (Fig. 2). The set of up-regulated genes includes HSP100, HSP90 and HSP70 gene families, known to contribute to the maintenance of cellular homeostasis during stress conditions in human, *Arabidopsis* and tomato [39–41]. Actually, HSP90 constitutes approximately 1-2% of the total protein content in eukaryotes suggesting complex interconnections with other key regulators and cochaperones in response to stress [42]. Moreover, HSP70 and HSP100 participate [43] with HSP90 during the proteome response to stress [42], also maintaining cellular homeostasis in physiological and stress conditions [39].

Despite small variations in their order of appearance, HSP90, HSP70 and HSP100 proteins are the first barrier in response to stress conditions in different organisms [39]. This observation motivates a computational strategy for uncovering the functionality of uncharacterized sHSPs in tomato, including the striking Solyc09g015000. For this purpose, we analyzed PPI networks in two independent heat shock experiments, (i) eight week tomato plants with up-regulated HsfA2 (Fig. 2a), and (ii) tomato protoplasts depleted in HsfA2 (Fig. 2b), where the first barrier of defense, mostly defined by high molecular weight HSPs, gets altered. In the former case, a protein network conformed by 15 nodes, almost all off them related to HSR (10 HSPs, HsfA2, and MBF1c), is observed (Fig. 2c). In the latter case, a small protein network conformed by eight nodes, all of them HsfA2 independent and up-regulated HSPs, can be observed. This network, which suggests the existence of alternative HSP regulatory mechanisms for HSR, includes the striking sHSP Solyc09g015000, three additional sHSPs of tandem duplication origin (Solyc08g062340, Solyc09g015020, and Solyc08g078700), another sHSP (Solyc05g014280), and three HSPs of high molecular weight (HSP100: Solyc03g115230, HSP90: Solyc03g007890, HSP70: Solyc04g011440) (Fig. 2d).

## Discussion

Promoter architecture plays a key role in the genome-wide transcriptional response of plants to different environmental factors [44, 45]. In particular, tandemly arrayed sHSP genes provide an opportunity to explore to what extent variations in their promoter architecture contribute to their functional diversity for environment adaptation [29, 46]. In this regard, we observed that although Solyc09g015000 is strongly up-regulated during fruit ripening, it is completely depleted from HSEs in its promoter region. Instead, the presence of a non-HSE CTAGA motif, hard to detect by PWM modeling due to its short length, can be be observed in its promoter region. Remarkably, this motif has been also observed in the promoter region of *Arabidopsis* MBF1c involved in thermotolerance [30], and in the promoter region of the MBF1c ortholog in tomato [27], suggesting that the CTAGA motif might be a valid DNA binding site for transcription factors enabling the response of certain sHSP genes, like Solyc09g015000, during fruit ripening.

To shed further light on the intriguing Solyc09g015000, a protein network conformed by HsfA2 independent proteins involved in HSR in tomato [47] was analyzed, and a protein network conformed by eight HSPs, five of them of the sHSP type, a single HSP70, a single HSP90, and a single HSP100, was observed (Fig. 2d). In this subnetwork, interactions between sHSPs are mediated by HSP70 and HSP90 proteins, both of them interacting with the single HSP100. Remarkably, these type of interactions can be also observed in acclimated *Arabidopsis* plants after severe heat shock (Additional file 2). Overall, this result suggests that Solyc09g015000 could be actually participating in the first barrier of stress response defined by HSP70, HSP90 and HSP100 proteins. In addition, this result suggests the existence of alternative, i.e., back-up, chaperone systems for stress response when part of the HSPs (70-90) paralogs gets altered, e.g., due to HsfA2 depletion. This plastic behavior of HSPs can be specifically appreciated in *Arabidopsis* thermotolerance experiments [48], where a small subset of chaperones (two mitochondrial, one cytosolic, one chloroplastic) and one HSP70 defines a characteristic mesh subnetwork in committed to survive plants (Additional file 2).

## Conclusions

Plants can cope with multiple stress situations. For each stimuli, specific stress response mechanisms are codified in the plant genome [49]. The diversity of expression patterns observed for the highly redundant family sHSP genes in tomato suggest that uncovering their promoter architecture may help to understand such mechanisms. For this purpose, stringent PWM models for the identification of the characteristic HSE motif in tomato sHSP genes were developed. As expected, HSEs were found in all, but one (Solyc09g015000), up-regulated linked to gene duplication sHSP gene promoters. Aiming to disentangle the functionality of such HSE exception, protein networks were additionally built revealing alternative stress response subnetworks, mostly conformed from sHSPs.

## Additional files

Additional file 1 HSFA1E and HSF21 TFBS associated to heat stress and ripening in tomato: building, quality controls and scanning for the promoter regions of sHSP gene family. (1) Gene IDs used to build HSFA1E and HSF21 PWMs. (2) Positive hits obtained with XXmotif tool. (3) PWM of the found TFBS in tomato and the corresponding known in *Arabidopsis* for heat stress (HSF1AE) and ripening (HSF21). (4) RSAT matrix-distrib output with theoretical distribution of weight scores (left) and corresponding decreasing cumulative distribution functions (right), for HSF1AE and HSF21 PWMs. (5) RSAT matrix-scan output table and location of the HSFA1E (red-squares) and HSF21 (blue-squares) motifs found in the promoter regions of 33 sHSPs in tomato. Differential sHSP gene expression during ripening according to [14] is indicated for up-regulation (UP), down-regulation (DOWN), not differentially expressed (NDE) and not expressed (NE) genes. (XLSX 137 kb)

Additional file 2 Protein IDs and output tables after STRING analysis. Protein IDs induced under similar experimental conditions of heat stress in (1) WT tomato plants (where HsfA2 is present), (2) tomato protoplasts (where HsfA2 was inhibited using RNAi) and (3) WT Arabidopsis plants, were used as input for STRING analysis. Overlapping nodes corresponding to HSP70, HSP90, HSP100 and sHSPs families, are indicated in bold in the output tables. (XLSX 123 kb)

## Abbreviations

EREs: Ethylene responsive elements
HSEs: Heat shock elements
HSPs: Heat shock proteins
HSR: Heat shock response
PWM: Position weight matrix
sHSPs: small heat shock proteins
TFBSs: Transcription factor binding sites
TSS: Transcription start site

## References

[1] Richter K, Haslbeck M, Buchner J (2010). The heat shock response: life on the verge of death,. Molecular cell.

[2] Al-Whaibi MH (2010). Plant heat-shock proteins: a mini review. J King Saud Univ - Science.

[3] Waters ER (1995). The Molecular Evolution of the Small Heat-Shock Proteins in Plants. Genetics.

[4] Scharf KD, Siddique M, Vierling E (2001). The expanding family of *Arabidopsis thaliana* small heat stress proteins and a new family of proteins containing alpha-crystallin domains (acd proteins). Cell Stress Chaperones.

[5] Park CJ, Seo YS (2015). Heat shock proteins: A review of the molecular chaperones for plant immunity. Plant Pathol J.

[6] Prasinos C, Krampis K, Samakovli D, Hatzopoulos P (2005). Tight regulation of expression of two Arabidopsis cytosolic Hsp90 genes during embryo development,. J Exp Bot.

[7] Faurobert M, Mihr C, Bertin N, Pawlowski T, Negroni L, Sommerer N, Causse M (2007). Major proteome variations associated with cherry tomato pericarp development and ripening. Plant Physiol.

[8] Li L, Wang X, Zhang X, Guo M, Liu T (2017). Unraveling the target genes of rin transcription factor during tomato fruit ripening and softening. J Sci Food Agric.

[9] Low D, Brandle K, Nover L, Forreiter C (2000). Cytosolic heat-stress proteins Hsp17.7 class I and Hsp17.3 class II of tomato act as molecular chaperones in vivo. Planta.

[10] Lawrence S, Cline K, Moore G (1997). Chromoplast development in ripening tomato fruit: identification of cdnas for chromoplast-targeted proteins and characterization of a cdna encoding a plastid-localized low-molecular-weight heat shock protein. Plant Mol Biol.

[11] Neta-Sharir I, Isaacson T, Lurie S, Weiss D (2005). Dual role for tomato heat shock protein 21: Protecting photosystem ii from oxidative stress and promoting color changes during fruit maturation. Plant Cell.

[12] Rizzon C, Ponger L, Gaut BS (2006). Striking similarities in the genomic distribution of tandemly arrayed genes in arabidopsis and rice. PLoS Comput Biol.

[13] Hanada K, Zou C, Lehti-Shiu MD, Shinozaki K, Shiu SH (2008). Importance of lineage-specific expansion of plant tandem duplicates in the adaptive response to environmental stimuli. Plant Physiol.

[14] Krsticevic FJ, Arce DP, Ezpeleta J, Tapia E (2016). Tandem Duplication Events in the Expansion of the Small Heat Shock Protein Gene Family in *Solanum lycopersicum* (cv. Heinz 1706). G3 (Bethesda).

[15] Yu J, Cheng Y, Feng K, Ruan M, Ye Q, Wang R, Li Z, Zhou G, Yao Z, Yang Y, Wan H (2016). Genome-wide identification and expression profiling of tomato hsp20 gene family in response to biotic and abiotic stresses. Front Plant Sci.

[16] Ouyang Y, Chen J, Xie W, Wang L, Zhang Q (2009). Comprehensive sequence and expression profile analysis of hsp20 gene family in rice. Plant Mol Biol.

[17] Sarkar NK, Kim Y-K, Grover A (2009). Rice shsp genes: genomic organization and expression profiling under stress and development. BMC Genomics.

[18] Guo M, Liu JH, Lu JP, Zhai YF, Wang H, Gong ZH, Wang SB, Lu MH (2015). Genome-wide analysis of the cahsp20 gene family in pepper: comprehensive sequence and expression profile analysis under heat stress. Front Plant Sci.

[19] Giorno F, Wolters-Arts M, Grillo S, Scharf KD, Vriezen WH, Mariani C (2010). Developmental and heat stress-regulated expression of hsfa2 and small heat shock proteins in tomato anthers. J Exp Bot.

[20] Kotak S, Larkindale J, Lee U, von Koskull-Döring P, Vierling E, Scharf KD (2007). Complexity of the heat stress response in plants. Curr Opin Plant Biol.

[21] Waters ER (2013). The evolution, function, structure, and expression of the plant shsps. J Exp Bot.

[22] Amin J, Ananthan J, Voellmy R (1988). Key features of heat shock regulatory elements. Mol Cell Biol.

[23] Akerfelt M, Morimoto RI, Sistonen L (2010). Heat shock factors: integrators of cell stress, development and lifespan. Nat Rev Mol Cell Biol.

[24] Bonner J, Ballou C, Fackenthal DL (1994). Interactions between dna-bound trimers of the yeast heat shock factor. Mol Cell Biol.

[25] Medina-Rivera A, Abreu-Goodger C, Thomas-Chollier M, Salgado H, Collado-Vides J, van Helden J (2011). Theoretical and empirical quality assessment of transcription factor-binding motifs. Nucleic Acids Res.

[26] Leibovich L, Paz I, Yakhini Z, Mandel-Gutfreund Y (2013). Drimust: a web server for discovering rank imbalanced motifs using suffix trees. Nucleic Acids Res.

[27] Fragkostefanakis S, Simm S, Paul P, Bublak D, Scharf K-D, Schleiff E (2015). Chaperone network composition in *Solanum lycopersicum* explored by transcriptome profiling and microarray meta-analysis. Plant Cell Environ.

[28] Luehr S, Hartmann H, Söding J (2012). The xxmotif web server for exhaustive, weight matrix-based motif discovery in nucleotide sequences. Nucleic Acids Res.

[29] Goyal RK, Kumar V, Shukla V, Mattoo R, Liu Y, Chung S, Giovannoni JJ, Mattoo AK (2012). Features of a unique intronless cluster of class i small heat shock protein genes in tandem with box c/d snorna genes on chromosome 6 in tomato (*Solanum lycopersicum*). Planta.

[30] Suzuki N, Sejima H, Tam R, Schlauch K, Mittler R (2011). Identification of the mbf1 heat-response regulon of arabidopsis thaliana. Plant J.

[31] Medina-Rivera A, Defrance M, Sand O, Herrmann C, Castro-Mondragon JA, Delerce J, Jaeger S, Blanchet C, Vincens P, Caron C, Staines DM, Contreras-Moreira B, Artufel M, Charbonnier-Khamvongsa L, Hernandez C, Thieffry D, Thomas-Chollier M, van Helden J (2015). RSAT 2015: Regulatory Sequence Analysis Tools. Nucleic Acids Res.

[32] Gupta S, Stamatoyannopoulos JA, Bailey TL, Noble WS (2007). Quantifying similarity between motifs. Genome Biol.

[33] O’Malley RC, Huang SS, Song L, Lewsey MG, Bartlett A, Nery JR, Galli M, Gallavotti A, Ecker JR (2016). Cistrome and epicistrome features shape the regulatory dna landscape. Cell.

[34] Turatsinze JV, Thomas-Chollier M, Defrance M, van Helden J (2008). Using RSAT to scan genome sequences for transcription factor binding sites and cis-regulatory modules. Nat Protoc.

[35] Pundir S, Martin MJ, O’Donovan C (2017). Uniprot protein knowledgebase. Methods Mol Biol.

[36] Szymanski J, Levin Y, Savidor A, Breitel D, Chappell-Maor L, Heinig U, Topfer N, Aharoni A (2017). Label-free deep shotgun proteomics reveals protein dynamics during tomato fruit tissues development. Plant J.

[37] Szklarczyk D, Morris JH, Cook H, Kuhn M, Wyder S, Simonovic M, Santos A, Doncheva NT, Roth A, Bork P, Jensen LJ, von Mering C. The string database in 2017: quality-controlled protein-protein association networks, made broadly accessible. Nucleic Acids Res. 2017;45(D1):362–8. 10.1093/nar/gkw937 PMC5210637 27924014

[38] Szklarczyk D, Franceschini A, Wyder S, Forslund K, Heller D, Huerta-Cepas J, Simonovic M, Roth A,Santos A, Tsafou KP, Kuhn M, Bork P, Jensen LJ, von Mering C. STRING v10: proteinŰprotein interaction networks, integrated over the tree of life. Nucleic Acids Res. 2015;43(Database issue): D447–52. 10.1093/nar/gku1003 PMC4383874 25352553

[39] Jacob P, Hirt H, Bendahmane A (2017). The heat-shock protein/chaperone network and multiple stress resistance. Plant Biotechnol J.

[40] Barah P, Naika MBN, Jayavelu ND, Sowdhamini R, Shameer K, Bones AM (2016). Transcriptional regulatory networks in *Arabidopsis thaliana* during single and combined stresses. Nucleic Acids Res.

[41] Scharf KD, Berberich T, Ebersberger I, Nover L (2012). The plant heat stress transcription factor (hsf) family: Structure, function and evolution?. Biochimica et Biophysica Acta.

[42] Krukenberg KA, Street TO, Lavery LA, Agard DA (2011). Conformational dynamics of the molecular chaperone hsp90. Q Rev Biophys.

[43] Mogk A, Kummer E, Bukau B (2015). Cooperation of hsp70 and hsp100 chaperone machines in protein disaggregation. Front Mol Biosci.

[44] Guan J-C, Yeh C-H, Lin Y-P, Ke Y-T, Chen M-T, You J-W, Liu Y-H, Lu C-A, Wu S-J, Lin C-Y (2010). A 9bp cis-element in the promoters of class i small heat shock protein genes on chromosome 3 in rice mediates l-azetidine-2-carboxylic acid and heat shock responses. J Exp Bot.

[45] Komarnytsky S, Borisjuk N, Setlow JK (2003). Functional Analysis of Promoter Elements in Plants. Genetic Engineering: Principles and Methods.

[46] Flagel LE, Wendel J (2009). Gene duplication and evolutionary novelty in plants. New Phytologist.

[47] Fragkostefanakis S, Mesihovic A, Simm S, Paupiere MJ, Hu Y, Paul P, Mishra SK, Tschiersch B, Theres K, Bovy A, Schleiff E, Scharf KD (2016). Hsfa2 controls the activity of developmentally and stress-regulated heat stress protection mechanisms in tomato male reproductive tissues. Plant Physiol.

[48] Echevarria-Zomeno S, Fernandez-Calvino L, Castro-Sanz AB, Lopez JA, Vazquez J, Castellano MM (2016). Dissecting the proteome dynamics of the early heat stress response leading to plant survival or death in arabidopsis. Plant Cell Environ.

[49] Baurle I (2016). Plant heat adaptation: priming in response to heat stress. F1000Res.

